# MiR-203 Targets to the 3′-UTR of SLUG to Suppress Cerebral Infarction-Induced Endothelial Cell Growth and Motility

**DOI:** 10.1155/2021/5597567

**Published:** 2021-03-01

**Authors:** Yunsong Li, Wei Bi, Bing Han, Tao Yuan, Long Shi, Yang Liu, Huanhuan Sun, Xueyan Li, Xiang Gao

**Affiliations:** ^1^Department of Vascular Surgery, Second Hospital of Hebei Medical University, Shijiazhuang 050000, China; ^2^Department of Vascular Surgery, Third Hospital of Hebei Medical University, Shijiazhuang 050000, China; ^3^Department of Vascular Surgery, Baoding No. 2 Central Hospital, Zhuozhou 072750, China

## Abstract

Cerebral infarction is one of the leading causes of death worldwide, in which angiogenesis plays a critical role. On the other hand, accumulating evidence has demonstrated that microRNAs (miRNAs) function as key modulators in the formation and progression of cerebral infarction. However, the molecular mechanisms of miRNAs underlying cerebral infarction-associated angiogenesis remain unclear. In the present study, we indicated that the expression of miR-203 was significantly downregulated in serum samples derived from patients with cerebral infarction and in mice brain samples following middle cerebral artery occlusion (MCAO) compared with healthy controls. *In vitro*, the expression of miR-203 was obviously downregulated in hypoxia-induced human umbilical vein vascular endothelial cells (HUVECs). Functionally, ectopic expression of miR-203 drastically suppressed HUVEC proliferation, invasion, and migration. In addition, SLUG, a zinc finger transcriptional repressor, was identified as a direct target of miR-203 and was negatively correlated with miR-203 expression in MCAO mice and in hypoxia-induced HUVECs. Furthermore, overexpression of SLUG reversed the inhibitory effect of miR-203 on proliferation, invasion, and migration abilities of HUVECs. Taken together, our research provides a novel insight of the miR-203-SLUG axis into cerebral infarction-associated endothelial behaviors and may offer a powerful therapeutic target of cerebral ischemia.

## 1. Introduction

Cerebral infarction is one of the leading causes of death for people over 60 years old worldwide [1–3]. Cerebral infarction frequently results in irreversible neurological deficits and is difficult to diagnose as powerful diagnostic markers are lacking. Although accumulating studies have demonstrated that a wide range of genes and signaling pathways are involved in cerebral ischemia, the precise molecular mechanisms remain not fully elucidated. Previous studies have proved that ischemic stroke can initiate angiogenesis to recover the oxygen and nutrient supply and facilitate functional recovery in damaged brain tissues [4–7]. However, the molecular mechanisms underlying cerebral infarction-related angiogenesis are not well documented.

miRNAs represent a class of endogenous, single-strand, small noncoding RNAs with approximately 22 nucleotides in length, which posttranscriptionally suppress target gene expression by targeting its 3′-untranslated region (3′-UTR) [8–10]. miRNAs have been widely reported to be implicated in multiple physiological and pathological processes, including cell proliferation, apoptosis, tissue homeostasis, organ development, carcinogenesis, inflammation, and ischemic diseases. Emerging evidence has indicated that miRNAs are of great importance in cerebral infarction. For instance, Cai et al. have reported that miR-146b-3p regulates the development and progression of cerebral infarction with diabetes through RAF1/P38MAPK/COX-2 signaling pathway [11]. On the other hand, miRNAs also play an essential role in angiogenesis and other biological behaviors of endothelial cells. For example, Zhao et al. have demonstrated that miR-124 aggravates failing hearts by suppressing CD151-facilitated angiogenesis in the heart [12]. However, limited research has paid attention to mechanisms of miRNAs underlying cerebral infarction-associated angiogenesis.

MiR-203 (also named miR-203a-3p) is a tumor suppressor miRNA playing an important role in various types of cancers and serving as an epidermis-specific miRNA essential for skin development. Much evidence has shown that miR-203 modulates cancer initiation and progression by targeting SLUG [13–16]. Moreover, miR-203 has been reported to regulate angiogenesis in cancers and the placenta [14, 17, 18] by targeting VEGFA, VEGFR2, and SLUG. However, little research has paid attention to the role of the miR-203-SLUG axis in ischemia, especially in cerebral infarction and cerebral infarction-related angiogenesis.

In the present study, we found that miR-203 was significantly downregulated in patients with cerebral infarction, MCAO mice, and hypoxia-induced HUVECs. Meanwhile, a reverse correlation between miR-203 and SLUG was observed in these samples. Importantly, overexpression of miR-203 in hypoxia-induced HUVECs suppressed cell proliferation, invasion, and migration, which could be reversed by ectopic expression of SLUG. Our findings not only demonstrate the function of the miR-203-SLUG axis in cerebral ischemia but also provide a putative therapeutic target for related diseases.

## 2. Materials and Methods

### 2.1. Patient Sample Collections

Patients with cerebral infarction (*n* = 18) were collected by the Department of Vascular Surgery, Second Hospital of Hebei Medical University from January 2017 to December 2017, which was approved by the Ethics Committee of Hebei Medical University. Sixteen healthy volunteers were enrolled as normal controls. Venous blood samples (5–10 ml) were collected from all the participants, which were centrifuged at 2000 rpm at 4°C for 30 min. Then the serum samples were isolated and stored at −80°C until analysis. Patients with other CNS diseases were excluded.

### 2.2. MCAO Mouse Model Constructions

Six- to eight-week-old male mice were obtained from Beijing Vital River Laboratory Animal Technology Co., Ltd. All animal procedures were approved by the Institutional Animal Care and Use Committee of Hebei Medical University. Mice were anesthetized with an intraperitoneal injection of a mixture of medetomidine hydrochloride (0.315 mg/kg), midazolam (2.0 mg/kg), and butorphanol tartrate (2.5 mg/kg), which was maintained at 37°C. Then, the cerebral ischemia was produced by intraluminal occlusion of the right middle cerebral artery using a nylon filament. The mice were randomly divided into two groups, with one group treated for 1 hour, the other for two hours. Later, the filament was withdrawn to allow reperfusion for 4 h. For the control group, the internal carotid artery was dissected without occlusion with a nylon filament. After that, all the animals were sacrificed with analgesics to obtain the brain tissues for further analyses.

### 2.3. Hypoxia-Induced HUVECs

The HUVECs were purchased from Life Technologies (Carlsbad, CA, USA) and cultured in Medium 200 with a low serum growth supplement. After being cultured in good condition with the medium containing 10% FBS (Gibco), 20 *μ*g/ml VEGF (Gibco), 100 U/mL penicillin, and 100 *μ*g/ml streptomycin, and the confluency being up to 80%, the cells for hypoxia models were incubated in the hypoxic medium in the absence of FBS and glucose, and the culture dish was put into a sterilized hypoxic box for 2 h, 4 h, and 6 h, with the concentration of oxygen kept less than 1% with 5% CO_2_ and 95% N_2_.

### 2.4. RNA Isolation and Quantitative RT-PCR

For mRNA detection, total RNA was isolated from the serum samples, brain tissues, or harvested cells by using TRIzol total RNA isolation kit (Tiangen, Beijing, China). Quantitative RT-PCR was performed using the One-Step SYBR^®^ PrimeScript™ RT-PCR kit (Takara, Dalian, China) on the Lightcycler Real-time PCR detection system (BioRad, Hercules, CA). GAPDH was used as an internal control. Primer sequences were as follows: 5′- ATGAGGAATCTGGCTGCTGT-3′ (SLUG, forward), 5′- CAGGAGAAAATGCCTTTGGA-3′ (SLUG, reverse), 5′- TCGGAGTCAACGGATTTGGT-3′ (GAPDH, forward), and 5′- TTGGAGGGATCTCGCTCCT-3′ (GAPDH, reverse). The TaqMan Human miRNA Assays were applied for miR-203 detection. Briefly, 5 ng of total RNA was reversely transcribed to cDNA with stem-loop primers by using the TaqMan miRNA Reverse Transcription Kit (Ambion, Carlsbad, California, USA). Quantitative real-time PCR (qRT-PCR) was carried out by TaqMan Universal PCR Master Mix. All PCR primers were from the TaqMan miRNA Assays. U6 RNA was used as an internal control.

### 2.5. Luciferase Reporter Assay

Reporter plasmid containing wild-type SLUG or mutant plasmid was transfected into HUVECs along with miR-203 or control mimic, using the FUGENE HD reagent (Roche, Mannheim, Germany) according to the manufacturer's protocol. Twenty-four hours later, cells were lysed with 100 *µ*l of Passive Lysis Buffer (Promega, Fitchburg, WI), and their Renilla luciferase levels were analyzed using the Dual Glo Luciferase Assay System (Promega). The firefly luciferase activity was used as an internal control.

### 2.6. Lentiviral Production

The cDNA of SLUG was cloned into the pSin4-EF2-IRES-Pur vector at the *SpeI* and *EcoRI* sites. The pSIN lentiviral system was used for ectopic expression of SLUG in HUVECs. The pSIN-SNAI, psPAX2, and pMD2G plasmids were cotransfected into HEK-293T cells using the Fugene HD reagent. The supernatant containing lentivirus was harvested and then filtered at 48 h after transfection.

### 2.7. Cell Proliferation Assay

Cell proliferation assay was performed using the MTT Cell Proliferation and Cytotoxicity Assay Kit (Beyotime, Shanghai, China) according to the manufacturer's manual. Briefly, HUVEC suspensions (2 × 10^4^ cells) were seeded into a 96-well cell culture plate. 20 *μ*l of MTT (5 mg/ml) was added to each well. Then, the plates were incubated at 37°C for 4 h to allow the MTT to react with viable cells to form formazan crystals. After that, the medium was removed, the formazan crystals were dissolved in 100 *µ*l of DMSO at 37°C for 15 min. The absorbance was measured by a microplate reader (Thermo Fisher Scientific, Waltham, MA, USA) at 570 nm.

### 2.8. Western Blot

Western blot was performed as previously described [19]. Rabbit polyclonal SLUG antibody was purchased from Abcam (Cat# 27568, Cambridge, MA, USA). Mouse monoclonal *β*-actin antibody was from Santa Cruz Biotechnology (Cat# 47778, Santa Cruz, CA, USA).

### 2.9. Invasion and Migration Assay

For invasion assay, 1 × 10^5^ cells were suspended into 100 *µ*l of fresh culture medium and seeded into the top chamber of a 24-well transwell insert (pore size: 8 *µ*m, BD Biosciences, Franklin Lakes, NJ, USA) precoated with 25 *µ*l of growth-factor reduced Matrigel (diluted into three volumes of serum-free culture medium). For migration assay, cells (1 × 10^5^) were suspended into 100 *μ*l of fresh medium and seeded in the top chamber of an insert, which was inserted into a 24-well plate. Five hundred *μ*l of culture medium with 10% FBS was added to the bottom chambers. Twenty-four hours later, the cell on the upper layer of the insert was removed by a cotton swab. The invading or migrated cells were fixed in 4% paraformaldehyde (Beyotime), stained with 0.1% crystal violet (Beyotime), and photographed under the Nikon inverted microscope. Staining was then dissolved with 10% acetic acid and quantified at a microplate reader at 570 nm.

### 2.10. Statistical Analysis

The statistical significance among the groups was determined by an unpaired Student's *t*-test. The correlation between miR-203 and SLUG was analyzed by the Pearson method. A *P*-value smaller than 0.05 was considered statistically significant.

## 3. Results

### 3.1. MiR-203 Expression Is Reversely Correlated with SLUG in Serum Samples Derived from Patients with Ischemic Infarction, Brain Tissues Derived from MCAO Mice, and Hypoxia-Induced HUVECs

**Figure 1:**
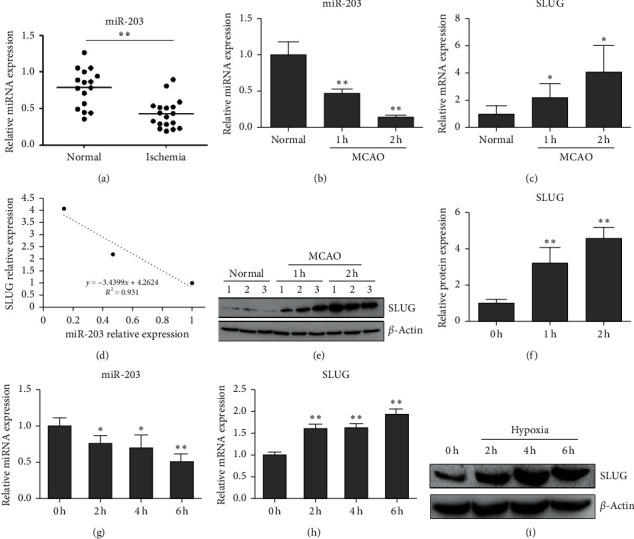
MiR-203 expression is downregulated in serum samples derived from patients with ischemic infarction, brain tissues from MCAO mice, and hypoxia-induced HUVECs, and its expression is negatively correlated with SLUG. (a) The expression of circulating miR-203 in patients with ischemic infarction compared with healthy control. (b) The expression of miR-203 in brain tissues of MCAO mice in 1 h and 2 h experimental groups compared with normal control. (c) SLUG expression in brain tissues of MCAO mice treated with 1h- and 2h-occlusion compared with normal control. (d) Correlation analysis of miR-203 and SLUG expression in brain tissues of MCAO mice. (e, f) Western blot to determine the protein level of SLUG in brain tissues of two groups of MCAO mice compared with normal control (*n* = 3). The relative protein level of SLUG was shown in (f). (g) miR-203 expression in hypoxia-induced HUVECs, compared with normal cells. (h) SLUG expression in hypoxia-induced HUVECs compared with normal cells. (i) Western blot analysis to evaluate the protein level of SLUG in hypoxia-induced HUVECs compared with normal cells. ^*∗*^*P* < 0.01, ^*∗∗*^*P* < 0.01 versus control.

Firstly, to evaluate the function of the miR-203-SLUG axis in cerebral infarction, we determined the expression level of circulating miR-203 in patients with ischemic infarction (*n* = 18) and healthy controls (*n* = 16) by using quantitative RT-PCR. We found that miR-203 was significantly downregulated in patients with cerebral infarction compared with healthy controls (*P*=0.0035, Figure 1(a)). We then established an MCAO model in mice and determined the expression levels of miR-203 and SLUG in brain tissues. Compared with the sham group, the expression of miR-203 was significantly reduced in both 1h- and 2h-occlusion groups (Figure 1(b)), while the expression of SLUG was notably elevated (Figures 1(c), 1(e) and 1(f)) and a reverse correlation between miR-203 and SLUG was observed (Figure 1(d)). Next, we examined miR-203 and SLUG expression in hypoxia-induced HUVECs. The results demonstrated that miR-203 was obviously downregulated in HUVECs with hypoxic treatment compared with normal cells (Figure 1(g)), while SLUG was upregulated (Figure 1(h) and 1(i)). These findings indicate that downregulation of miR-203 and upregulation of SLUG may be attributed to ischemia.

### 3.2. SLUG Is Direct Target of MiR-203 in HUVECs

**Figure 2:**
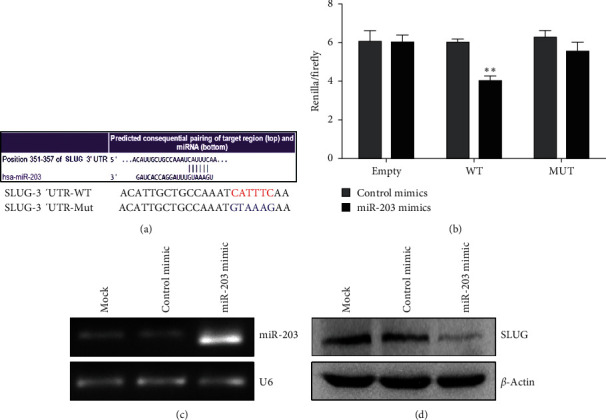
SLUG is a direct target of miR-203 in HUVECs. (a) Schema of miR-203 binding sites within the SLUG 3′UTR. Mutated sites were shown in blue. (b) The psiCHECK-2 sensor plasmids containing the SLUG 3′UTR or mutated 3′UTR were cotransfected in HUVECs with miR-203 mimic or control mimic. Overexpression of miR-203 selectively decreased the luciferase expression compared to controls. No downregulation of luciferase was observed for the negative control or mutated plasmids with miR-203 overexpression. (c) Semiquantitative RT-PCR to determine the expression of miR-203 in HUVECs transfected with miR-203 mimic compared with control. (d) Western blot to determine the protein level of SLUG in HUVECs transfected with miR-203 mimic compared with control. ^*∗∗*^*P* < 0.001 versus control.

To elucidate whether miR-203 directly targets SLUG in HUVECs, we constructed a luciferase reporter containing the wild-type SLUG 3′-UTR or mutant plasmid for miR-203 binding sites (Figure 2(a)) and performed a luciferase report assay in HUVECs. The results indicated that cotransfection of miR-203 mimic suppressed the luciferase activity of the reporter with wild-type SLUG 3′UTR but failed to suppress that with mutant reporter plasmid (Figure 2(b)). In addition, transfection of miR-203 mimic in HUVECs (Figure 2(c)) significantly inhibited the protein level of SLUG, which was determined by using Western blot analysis (Figure 2(d)). These results demonstrate that endogenous SLUG in HUVECs is directly targeted by miR-203.

### 3.3. The MiR-203-SLUG Axis Functions as a Key Regulator in Hypoxia-Induced Cell Proliferation and Migration in HUVECs

**Figure 3:**
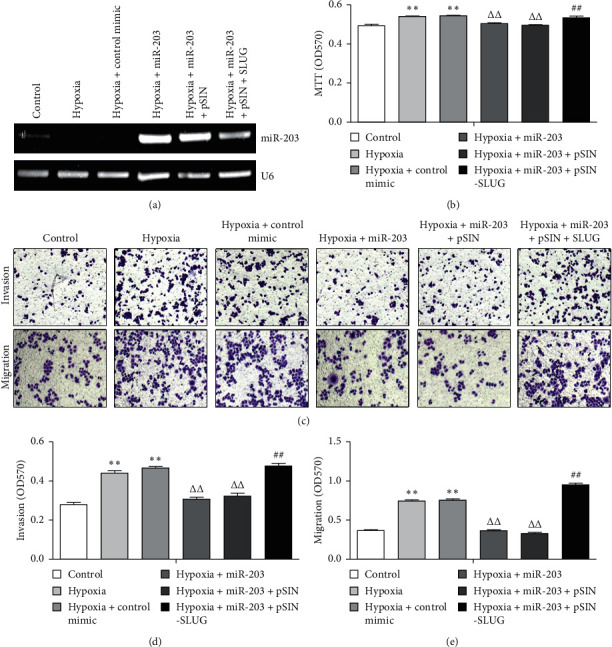
The miR-203-SLUG axis functions as a key regulator in hypoxia-induced cell proliferation, invasion, and migration. (a) Semiquantitative RT-PCR to determine the expression of miR-203 in HUVECs with indicated treatment. (b) MTT assay to determine the cell proliferation rate of HUVECs with indicated treatment. (c–e) Transwell invasion and migration assays to determine the invasion and migration abilities of HUVECs with indicated treatment. Cells invading or migrating to the bottom side of the upper chamber were stained with crystal violet, dissolved with acetic acid, and quantified at OD570 (D and E). ^*∗∗*^*P* < 0.001 versus control; ^ΔΔ^*P* < 0.001 versus hypoxia + control mimic; ^##^*P* < 0.001 versus hypoxia + miR-203 mimic.

It is well known that cell proliferation and migration play an important role in hypoxia-induced angiogenesis. We next determined the effect of miR-203-SLUG in hypoxia-induced cell proliferation and migration in HUVECs. The MTT assay demonstrated that hypoxic treatment induced HUVEC proliferation (Figure 3(b)), which was suppressed by transfection of miR-204 mimic (Figures 3(a) and 3(b)). However, when we overexpressed SLUG in miR-203-transfected cells, hypoxic treatment reelevated HUVEC proliferation again (Figure 3(b)). A similar phenomenon was seen in the invasion and migration assay whereby the HUVECs transfected with miR-203 suppressed hypoxia-induced cellular invasion and migration, which was erased by ectopic expression of SLUG (Figures 3(c)–3(e)). Taken together, these findings suggest that the miR-203-SLUG axis functions as a key modulator in the biological behavior of HUVECs, and thus contributes to angiogenesis in cerebral infarction.

Moreover, to determine the effect of SLUG on the biological behaviors of HUVEC induced by hypoxia, we silenced the expression of SLUG in hypoxia-treated HUVECs (Figure S1A). The results demonstrated that the silence of SLUG drastically attenuated the proliferation and migration rates of HUVECs induced by hypoxia (Figures S1B and S1C).

## 4. Discussion

Deregulation of miRNAs and transcription factors contributes to a wide range of pathologic diseases, including ischemia. In the present study, we found that the expression of miR-203 was drastically downregulated in patients with ischemic infarction, MCAO mice and hypoxia-treated HUVECs compared with normal controls, while SLUG was upregulated. Moreover, we demonstrated that miR-203 suppressed HUVEC proliferation, invasion, and migration via targeting SLUG. These results suggest that the miR-203-SLUG axis is of great importance in the pathogenesis of cerebral infarction.

In this study, we focused on miR-203, as this miRNA has been previously reported as a tumor suppressor miRNA in carcinogenesis associated with tumor angiogenesis. Several studies have also demonstrated that miRNAs play a critical role in the nervous system. Tripathi et al. revealed that riboflavin treatment increased miR-203 expression, which in turn inhibited the c-Jun expression and increased neuronal cell survival [20]. Yang et al. reported that miR-203 negatively regulated ischemia-induced microglia activation by targeting MyD88, an important adapter protein involved in most Toll-like receptors (TLRs) and interleukin-1 receptor (IL-1R) pathways [21]. For the relationship between miR-203 and ischemia, only one literature reported that miR-203 functioned as a regulator in acute kidney injury, in which Aldosterone induced NRK-52E cell apoptosis in acute kidney injury via rno-miR-203 hypermethylation and Kim-1 upregulation [22]. In the present study, we systematically identified the expression, function and underlying mechanisms of miR-203 in cerebral infarction.

On the other hand, no literature has demonstrated the role of SLUG in the nervous system and ischemia. Only one study implicated snail as a potential target molecule in cardiac fibrosis after I/R injury [23]. In that paper, Lee et al. demonstrated that I/R injury to mouse hearts significantly increased the expression of snail. Importantly, the cell source of snail induction is endothelial cells. When snail was overexpressed in endothelial cells, they underwent endothelial-to-mesenchymal transition. Snail overexpression-mediated EMT-like cells noticeably stimulated trans-differentiation of fibroblasts to myofibroblasts. The injection of a peroxisome proliferator-activated receptor-*γ* (PPAR-*γ*) agonist, a selective snail inhibitor, remarkably suppressed collagen deposition and cardiac fibrosis in mouse I/R injury and significantly improved cardiac function and reduced snail expression *in vivo*. In our present study, we also demonstrated that ischemia and hypoxia-induced SLUG expression in brain tissues and HUVECs. Overexpression of SLUG induced cell invasion and migration in HUVECs.

In order to further verify that the cytological mechanism of the reverse correlation between miR-203 and SLUG is related to cell proliferation, invasion, and migration, the study established an in vitro hypoxia-induced HUVECs model and confirmed cell proliferation, invasion, and migration, which were reversed by ectopic expression of SLUG, in such a hypoxic condition. The above research findings further support the role of the miR-203-SLUG axis in the angiogenesis of cerebral infarction. Our study first elucidates the role of SLUG in cerebral infarction.

## Data Availability

The data used to support the finding of this study are available from the corresponding author upon request.

## Supplementary Materials

Supplementary Materials Figure S1: Silencing of SLUG attenuates the proliferation and migration rates of HUVECs induced by hypoxia. (a) Western blot to determine the protein level of SLUG in indicated groups. (b) MTT assay to determine cell proliferation rate of HUVECs with indicated treatment. ^*∗∗*^*P* < 0.001 versus control; ^##^*P* < 0.001 versus hypoxia + Ctrl siRNA. (c) Transwell migration assays to determine the migration abilities of HUVECs with indicated treatment.
