# Supplementary material for: MiR-203 Targets to the 3′-UTR of SLUG to Suppress Cerebral Infarction-Induced Endothelial Cell Growth and Motility
Source: Evid Based Complement Alternat Med. 2021 Mar 1;2021:5597567. doi: 10.1155/2021/5597567 (PMC7943304; doi:10.1155/2021/5597567)

**Figure S1 Silencing of SLUG attenuates the proliferation and migration rates of HUVECs induced by hypoxia.** (A) Western blot to determine the protein level of SLUG in indicated groups. (B) MTT assay to determine cell proliferation rate of HUVECs with indicated treatment. ^**^*P* < 0.001 versus control; ^##^*P* < 0.001 versus Hypoxia + Ctrl siRNA. (C) Transwell migration assays to determine the migration abilities of HUVECs with indicated treatment.


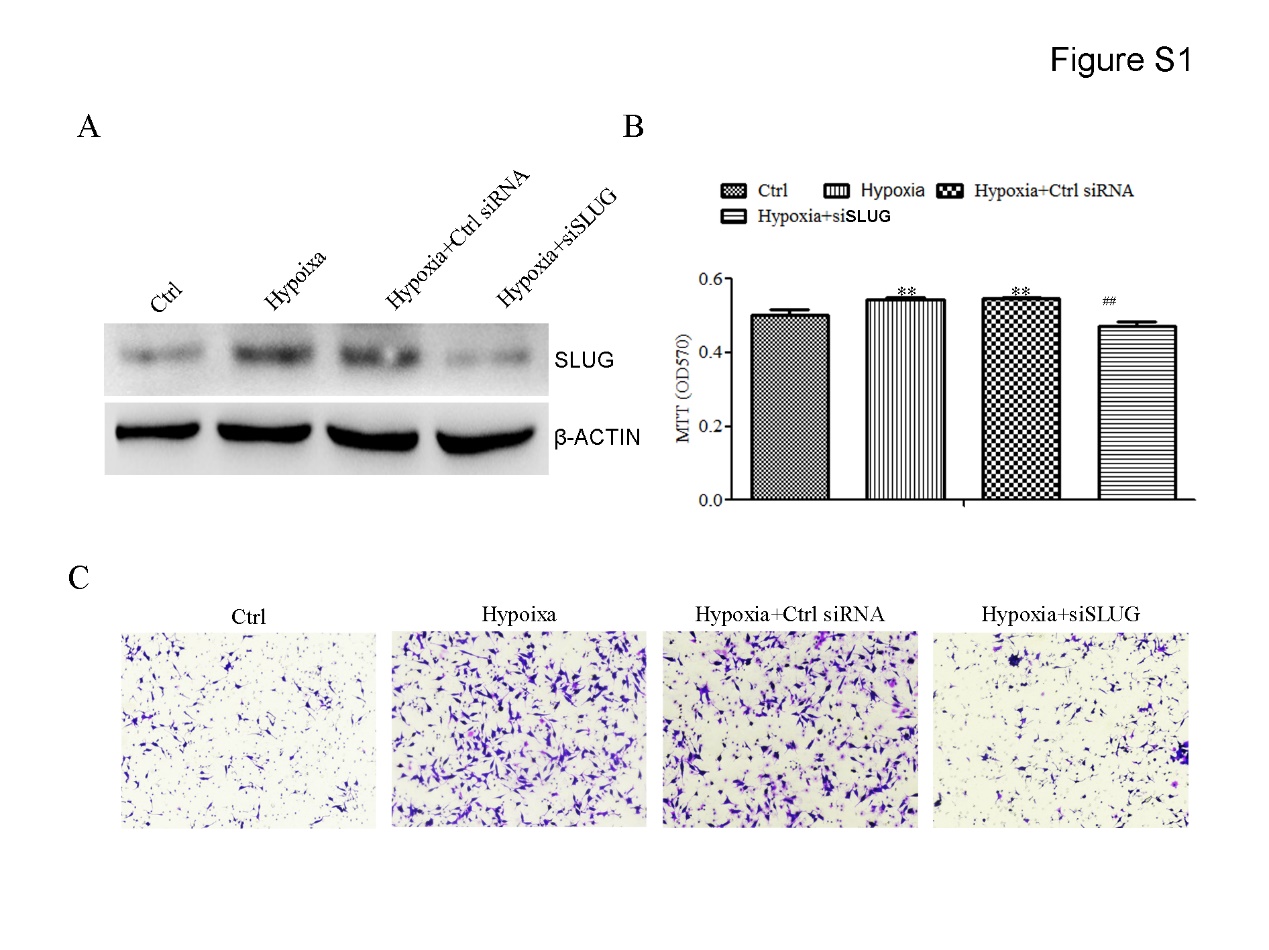

Supplement: Supplementary Materials — Figure S1: Silencing of SLUG attenuates the proliferation and migration rates of HUVECs induced by hypoxia. (a) Western blot to determine the protein level of SLUG in indicated groups. (b) MTT assay to determine cell proliferation rate of HUVECs with indicated treatment. ∗∗P < 0.001 versus control; ##P < 0.001 versus hypoxia + Ctrl siRNA. (c) Transwell migration assays to determine the migration abilities of HUVECs with indicated treatment. [file 5597567.f1.docx]
